# HARE: Unifying the Human Activity Recognition Engineering Workflow

**DOI:** 10.3390/s23239571

**Published:** 2023-12-02

**Authors:** Orhan Konak, Robin van de Water, Valentin Döring, Tobias Fiedler, Lucas Liebe, Leander Masopust, Kirill Postnov, Franz Sauerwald, Felix Treykorn, Alexander Wischmann, Hristijan Gjoreski, Mitja Luštrek, Bert Arnrich

**Affiliations:** 1 Hasso Plattner Institute, University of Potsdam, 14482 Potsdam, Germany; robin.vandewater@hpi.de (R.v.d.W.); valentin.doering@student.hpi.uni-potsdam.de (V.D.); tobias.fiedler@student.hpi.uni-potsdam.de (T.F.); lucas.liebe@student.hpi.uni-potsdam.de (L.L.); leander.masopust@student.hpi.uni-potsdam.de (L.M.); kirill.postnov@student.hpi.uni-potsdam.de (K.P.); franz.sauerwald@student.hpi.uni-potsdam.de (F.S.); felix.treykorn@student.hpi.uni-potsdam.de (F.T.); alexander.wischmann@student.hpi.uni-potsdam.de (A.W.); bert.arnrich@hpi.de (B.A.); 2 Faculty of Electrical Engineering and Information Technologies, Ss. Cyril and Methodius University in Skopje, 1000 Skopje, North Macedonia; hristijang@feit.ukim.edu.mk; 3 Department of Intelligent Systems, Jožef Stefan Institute, 1000 Ljubljana, Slovenia; mitja.lustrek@ijs.si

**Keywords:** human activity recognition, multimodal classification, privacy preservation, real-time classification, sensor placement

## Abstract

Sensor-based human activity recognition is becoming ever more prevalent. The increasing importance of distinguishing human movements, particularly in healthcare, coincides with the advent of increasingly compact sensors. A complex sequence of individual steps currently characterizes the activity recognition pipeline. It involves separate data collection, preparation, and processing steps, resulting in a heterogeneous and fragmented process. To address these challenges, we present a comprehensive framework, HARE, which seamlessly integrates all necessary steps. HARE offers synchronized data collection and labeling, integrated pose estimation for data anonymization, a multimodal classification approach, and a novel method for determining optimal sensor placement to enhance classification results. Additionally, our framework incorporates real-time activity recognition with on-device model adaptation capabilities. To validate the effectiveness of our framework, we conducted extensive evaluations using diverse datasets, including our own collected dataset focusing on nursing activities. Our results show that HARE’s multimodal and on-device trained model outperforms conventional single-modal and offline variants. Furthermore, our vision-based approach for optimal sensor placement yields comparable results to the trained model. Our work advances the field of sensor-based human activity recognition by introducing a comprehensive framework that streamlines data collection and classification while offering a novel method for determining optimal sensor placement.

## 1. Introduction

As part of pervasive computing, sensor-based human activity recognition (HAR) involves the classification of time series data produced by inertial measurement units (IMUs). IMUs primarily capture and measure quantities, such as acceleration and angular velocity, generating discrete temporal data. These data can be segmented into windows and classified, typically utilizing machine learning (ML) models. By leveraging these models, the activity recognition system can classify various activities based on the analyzed patterns within the time series data. The range of movements encompasses both low-level activities, such as walking and standing, as well as high-level activities that involve combinations of multiple low-level activities. HAR demonstrates promising applications across diverse domains, including healthcare, sports, and smart environments [1,2,3]. Similar to other research in ML, achieving reliable classification results in HAR necessitates a well-structured data pipeline encompassing qualitative data collection, preparation, and classification stages [4]. Each step within this pipeline is crucial in influencing the final classification outcome. Traditional approaches involve a fragmented and complex process, from data collection to processing, often leaving researchers grappling with disjointed methodologies. None of the available solutions adequately cater to the researchers’ needs for data acquisition, synchronization, anonymization, and enrichment with 2D landmarks of the users’ body, enabling a multimodal classification approach in an integrated software environment. Furthermore, there is a lack of solutions that guide optimal IMU placement for improved classification results prior to conducting a study on specific activities. Additionally, an integrated solution incorporating human-in-the-loop feedback, allowing users to receive real-time activity feedback on specific activities and retrain a personalized model based on their specific execution patterns, is currently absent. These gaps highlight the necessity for a comprehensive framework that addresses these critical aspects and empowers researchers in their endeavors [5].

**Figure 1:**
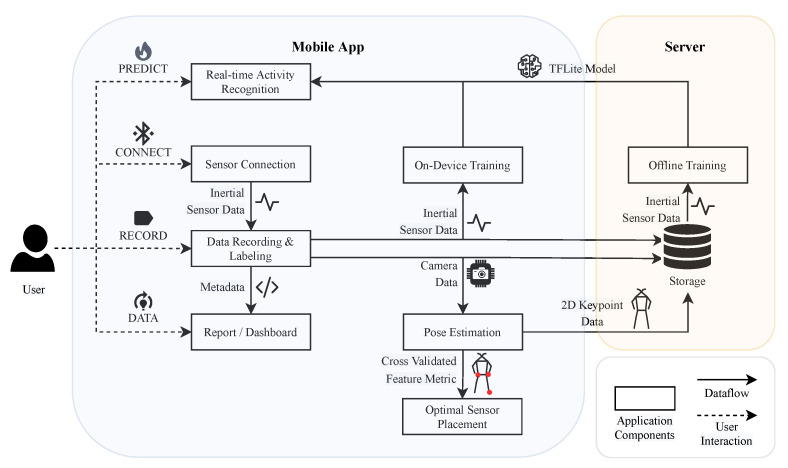
The application’s architecture can be divided into two parts—mobile app and server—to ensure efficient data handling and processing. Mobile app: The app comprises different views depicted as rectangles, each important to conduct a study for human activity recognition. The arrows illustrate the dataflow, which can be of different data modalities. Server: The collected data can be uploaded to a server, and a lightweight deep learning model can be trained and embedded into the app.

To bridge this gap in human activity recognition, we introduce HARE (human activity recognition engineering), a comprehensive framework that revolutionizes the traditional HAR process as illustrated in Figure 1. HARE stands out by offering a holistic approach, catering to essential researcher needs ranging from data collection and synchronization to advanced privacy-preserving techniques using 2D body landmarks. This integration facilitates a multimodal classification approach, enhancing the analysis and recognition of human activities. HARE’s innovation lies in its ability to efficiently handle all aspects of HAR data, ensuring seamless synchronization between multiple IMUs and video data. This synchronization facilitates label refinement and respects privacy by converting video data into 2D human pose estimations. Additionally, HARE introduces a novel sensor placement strategy based on these pose estimations, optimizing classification outcomes while reducing the reliance on IMUs. The framework’s flexible model training, offering both on-device and server-based options, its real-time feedback capabilities, and privacy-preserving adjustments on the device set it apart from existing methods in the field.

To assess the effectiveness of HARE, we employed a two-fold evaluation approach. Firstly, we utilized publicly available datasets commonly used in sensor-based HAR. Secondly, we conducted a controlled laboratory study involving ten subjects who performed nursing activities. The combination of existing datasets and a proof-of-concept study allows us to evaluate the performance and applicability of the HARE framework comprehensively. The evaluation allowed us to assess HARE’s performance in data collection, labeling, sensor placement, and real-time feedback, as well as its ability to preserve privacy during these processes. Through our evaluation, we found that multimodality increased the F1 score by up to 4.4% and on-device training by up to 58.7%. Moreover, HARE’s sensor placement recommendations demonstrated similarity to the optimal placements suggested by the best-performing deep learning model, specifically a CNN-LSTM architecture. Our findings demonstrate the effectiveness of HARE in addressing the current challenges in the field of HAR.

Our research makes the following contributions to the field of HAR:

- Combining synchronized data collection from wearable devices, a mobile phone camera, and 2D keypoints from pose estimation. This coordination enhances robust data acquisition and supports a multimodal classification approach to HAR from IMUs and 2D keypoints.
- Presenting an on-device working lightweight method using 2D pose estimation for determining optimal sensor placement, requiring only 500 data points, even from publicly available video footage of target activities.
- Accommodating diverse classification tasks using wearable, video, or skeleton inputs, with options to modify underlying classification and landmark detection models for specific cases, like face, eye, or hand recognition.
- Employing transfer learning for on-device training, which preserves data privacy and enhances model performance. The integration of active learning, demonstrated through a returned confusion matrix of each activity’s performance, supports continual data recollection and model refinement.
- Providing instant activity recognition and detailed statistics on person-specific and activity-based metrics. These insights can enhance business processes and support further research.

In the following sections of this paper, we provide a detailed roadmap of our study. Section 2 offers an in-depth exploration of related work, comparing the distinct features of HARE with other frameworks. In Section 3, we delve into HARE’s various features and functionalities, offering a comprehensive understanding of its core components. Moving on to Section 4, we present our evaluation of HARE’s capabilities across diverse datasets, including a self-recorded dataset capturing nursing activities. In Section 5, we shed light on our findings and their implications. Finally, in Section 6, we draw our conclusions, encapsulating the essence of our work and its contributions to HAR.

## 2. Related Work

Sensor-based HAR utilizing wearable sensors boasts diverse applications, extending from healthcare to surveillance [6,7,8]. HAR frameworks present a myriad of opportunities, serving various purposes such as monitoring activity levels to prevent injuries or for documentation [9,10,11,12]. Moreover, they can be used in threat detection and prevention [13]. The extensive array of potential applications has given rise to a substantial body of literature, encompassing a variety of research sub-domains within HAR [14,15,16,17,18]. While previous approaches have made significant contributions to these sub-areas, our approach seeks to bridge the gaps and address the limitations present in the existing literature. We provide an end-to-end solution for HAR tasks that can be applied to any area of interest where physical activities are present. To provide a comprehensive overview of the existing research, this section examines the current state-of-the-art techniques for HAR data collection and classification, as well as existing approaches to optimizing sensor placement for improved classification accuracy. In addition, we explore the current landscape of privacy-preserving on-device approaches for HAR. Throughout this section, we highlight the strengths and limitations of the existing approaches and compare them to HARE.

### 2.1. Data Acquisition and Labeling

Data collection and labeling in sensor-based HAR are significant challenges [19]. Existing research studies, such as those conducted in [20], have attempted to address these issues, highlighting the impact of additional data on performance and proposing methods to predict the performance improvement of HAR models after collecting additional data. However, these methods often encounter limitations when synchronizing data from multiple standalone IMU devices or collecting video recordings alongside IMU data for relabeling and quality assurance. The labor-intensive, time-consuming, and error-prone nature of data collection in HAR was emphasized in [5]. They proposed practical guidelines for efficient data collection based on control variable experiments involving over 240 subjects. Their insights could be further enhanced by leveraging synchronized multimodal data collection technology.

HARE offers a comprehensive and promising solution that addresses the challenges of data collection and labeling. It leverages real-time pose estimation from video recordings to facilitate data anonymization and relabeling, which mitigates privacy concerns associated with video data. Beyond this, HARE integrates capabilities for synchronized data collection from multiple standalone IMU devices and video recording. This enables a single or multimodal real-time classification approach not confined to a specific area of interest.

### 2.2. Sensor Placement

The issue of sensor placement arises as a crucial aspect when conducting studies in HAR. The classification accuracy heavily relies on the data received, which varies based on the location and number of sensors employed for different body parts [21]. Previous research indicates optimal results are achieved by placing sensors on the chest, ankles, and thighs [1]. Moreover, studies suggest simultaneously utilizing accelerometers on both the upper and lower torso significantly enhances activity recognition precision [22,23]. In a study examining accelerometer placement for categorizing physical activities and estimating energy expenditure in older adults [24], five different body positions were compared: wrist, hip, ankle, upper arm, and thigh. The study concludes that careful consideration of accelerometer placement is essential for optimizing the accuracy of HAR. In [25], the impact of sensor position on HAR system performance is discussed. Given a fixed number of sensors, the authors propose an optimization scheme to determine the optimal sensor position from a range of possible locations. By employing virtual sensor data and leveraging low-cost access to the training dataset, the system enables accurate decisions regarding sensor position selection through feedback mechanisms. There is also a study that investigates the optimal sensor placement by leveraging keypoints derived from pose estimations [26]. This research explores the correlation between body landmarks and the positioning of sensors, aiming to enhance the accuracy and performance of sensor-based HAR systems.

Building upon the approach introduced in [26], we extend and elaborate on the methodology by examining its effectiveness across various datasets and models, encompassing a broader range of activities. In contrast to existing approaches, our method eliminates the need for a physical sensor setup, instead relying on human pose estimations derived from either self-recorded videos or existing videos of the target activities within HARE. This approach significantly reduces the setup and calibration efforts typically associated with HAR, which improves user experience. Furthermore, our technique entails less computational complexity than classical approaches involving extensive training and testing with large datasets, enabling the use of mobile devices without external computing resources. This streamlined approach enhances efficiency and offers a practical solution for real-world applications.

### 2.3. Real-Time Feedback

Real-time activity recognition [27] has emerged as a crucial topic across numerous fields due to its potential to facilitate instant feedback and action, such as in healthcare [28,29,30]. The relevance has prompted a wealth of research studies, each contributing to the collective understanding and development in this field. In [31], they leverage the array of sensors in mobile devices to develop a user-independent, real-time activity recognition system using a combination of convolutional neural networks and statistical features. In [32], a novel deep learning model—the bidirectional-gated recurrent unit-inception using IMUs—was introduced. The model’s accuracy and robustness were evaluated on three datasets, demonstrating superior performance while using fewer sensors. In [33], an unsupervised online domain adaptation algorithm for smartphone accelerometers, addressing the challenge of personalizing models to new users and changing motion patterns was proposed. The authors’ solution involves real-time, incremental alignment of feature distributions across all subjects in hidden neural network layers, with user-specific mean and variance statistics. In [34] a computationally efficient deep learning method, specifically a conditionally parameterized convolutional neural network for real-time HAR on mobile and wearable devices was introduced. The proposed method effectively balances accuracy and computational cost.

Our proposed approach provides several additional features that make it particularly well-suited for use in real-world scenarios, unlike the aforementioned approaches, which have focused primarily on accuracy and computational complexity. For example, our model is both adaptable and uses less computing power, which allows it to be trained on-device and run efficiently on resource-constrained devices. Furthermore, we provide accuracy on all activities performed, which gives the opportunity to recollect data on poorly recognized activities. In addition, we include metadata statistics on the performed activities and persons, which provides the possibility for further analysis and process mining. These features are an improvement upon previous work and highlight its potential for practical use cases.

### 2.4. Privacy-Preserving On-Device Approaches

Privacy is a major concern in the field of HAR, where the collection and processing of personal data are an integral part of the process [35]. Several existing approaches, such as federated learning, differential privacy, and homomorphic encryption, have been proposed to address this issue and enable the training of HAR models on personal devices without compromising privacy [36,37]. However, challenges remain in ensuring the fidelity and security of the collected data. To address these challenges, recent research has made use of on-device learning and inference. In [38,39], models that support on-device incremental learning and inference—leading to a significant efficiency boost during inference—were proposed. A dynamic active learning-based approach that selects informative samples and identifies new activities to improve performance and reduce annotation cost was proposed in [35]. Further, in [40], a way to minimize the task of labeling data through an active learning approach is explored. These approaches allow for adaptive daily activity analysis and the detection of novel activities and patterns, with experimental results demonstrating their effectiveness on synthetic and benchmark datasets.

HARE stands out in this facet by offering on-device training aided by pre-trained models, which ensures that data are not leaving the device and thus maintains data privacy. Additionally, HARE provides a confusion matrix that encourages the collection of new data on poorly recognized movements, fostering active learning and improving performance.

### 2.5. Multimodal Classification Approaches

Building upon the work introduced in [26], we delve into multimodal HAR, which has gained significant attention in recent years. Integrating multiple sensory data sources offers the potential for more accurate and robust activity recognition compared to unimodal approaches [41,42]. For instance, in [43], the MMHAR-EnsemNet approach was proposed, which leverages four different modalities for sensor-based HAR and has been evaluated on two standard benchmark datasets. Previous studies, such as [44], have also explored methods for fusing and combining different representations of sensor data using deep convolutional neural networks, achieving promising results.

In contrast to these multimodal approaches, HARE takes a unique approach by utilizing a single device to collect data from IMUs and videos. These data are then transformed in real-time into 2D human pose estimations, resulting in an inherently given multimodal data stream that facilitates recording and classification processes. This distinctive methodology distinguishes HARE from existing multimodal approaches in sensor-based HAR.

HARE represents a significant advancement for the HAR community. Several studies have investigated some features independently to a limited extent. However, such studies remain narrow in focus as they cover just one aspect. We highlight the need for the unification of the features. HARE combines all necessary features for sensor-based HAR in one application and enriches it with newly developed functionalities. It offers a more comprehensive set of features, including support for a wide range of sensors, synchronized multimodal recording and classification, real-time classification, on-device training, and active learning. The framework is highly adaptable and, therefore, a versatile tool for a wide range of use cases. A comprehensive comparison of HARE with other tools and research methods in the HAR field, showcasing the unique and enriched functionalities, is shown in Table 1.

## 3. Application Functionalities

**Figure 2:**
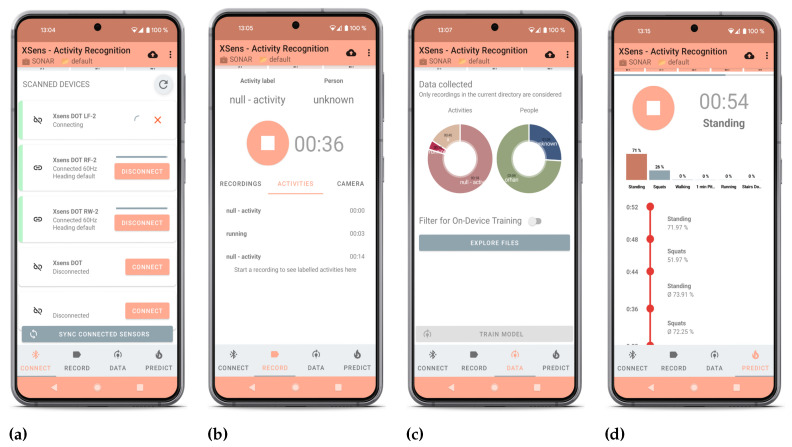
Different functionalities of the application highlight the versatility of HARE. (**a**) Connection overview for connecting and synchronizing the Xsens DOT sensors. (**b**) Recording interface for saving inertial, camera, and pose estimation data together with labeling activities in each timestamp. (**c**) Data dashboard allows exploring the recorded data. (**d**) Prediction overview allows real-time activity recognition for the incoming data.

As depicted in Figure 2, HARE integrates several essential features for HAR, including (1) data collection, (2) labeling and label refinement, (3) anonymization, (4) pose estimation, (5) optimal sensor placement, (6) multimodal HAR, (7) meta-analysis, (8) offline training, and (9) on-device active learning. The framework is developed for Android operating systems, specifically in Android 13, the latest version at the time of writing. Designed using TensorFlow Lite (TFLite), HARE benefits from TFLite’s operator versioning schema, which ensures backward compatibility (allowing newer implementations to handle older model files) and forward compatibility (enabling older implementations to handle new model files, provided no new features are used) [54]. This approach guarantees robust functionality across various Android devices and versions. We have rigorously tested HARE on various models, including Google Pixel Phones 6 and 7 Pro, confirming its adaptability to different hardware configurations. The Google Pixel 6 features a 6.4-inch display, 8 GB LPDDR5 RAM, and 128 GB storage, while the Google Pixel 7 Pro comes with a 6.7-inch display, 12 GB LPDDR5 RAM, and 256 GB storage. Both devices are equipped with Google’s Tensor processors and support high-resolution video recording capabilities, essential for the app’s pose estimation functionality. While initially developed for these models, the underlying technology in HARE is flexible enough for potential compatibility with a wide range of Android devices.

### 3.1. Architecture and Key Components

HARE’s architecture revolves around a single MainActivity, containing four primary screens, as depicted in Figure 2, managed by distinct packages and classes. This modular structure enhances the app’s functionality, with each screen dedicated to specific tasks such as managing sensor connections, data recording, and label management. The ConnectionScreen class, interfaces with Xsens DOT sensors by Movella in Enschede, Netherlands, utilizing the Xsens DOT SDK for seamless sensor management and data collection.

The following libraries and modules integrated into the app are instrumental in its functionality:

- Xsens™ DOT ([55]): Guides the integration of Xsens DOT sensors.
- WebDAV Client ([56]): Enables data storage on WebDAV cloud.
- Charts Library ([57]): Facilitates on-device data visualization.
- Tree View Library ([58]): Assists in organizing and presenting hierarchical data.

### 3.2. Connection

Due to its compact size and lightweight design, we opted to utilize the Xsens™ DOT sensor as a standalone device for unobtrusive data recording. We developed our framework using the Xsens DOT Android software development kit (SDK version v2020.4). This SDK enabled functionalities such as scanning, connecting, and receiving data seamlessly. The Xsens DOT sensor communicates with the host device via Bluetooth for data transmission. While there is no specific connection limit in the Xsens DOT SDK services, the maximum number of sensors that can be connected simultaneously depends on the central devices’ hardware and operating system constraints. In the case of Android, up to seven sensors can be connected. The output rate for real-time streaming can be adjusted, ranging from 1 Hz to 60 Hz, while the recording mode supports rates up to 120 Hz. All sensors are time-synchronized after the initial synchronization process. Sensor connection and synchronization are achieved within 3 to 5 s. The transmitted data from the Xsens DOT sensor includes calibrated orientation data (quaternion), inertial data, and magnetic field data. This comprehensive set of information allows for accurate and precise analysis of human movements.

### 3.3. Recording

Connecting the IMUs to the app enables data recording with various sensor data outputs, including quaternions, (free) acceleration, angular velocity, and magnetic field normalized to the earth’s field strength. The raw data from these sensors, including the quaternion data representing sensor orientation, are used directly without extensive preprocessing. The app provides functionality to adjust the output rates of these sensors based on the requirements of the activity recognition task.

For video data, frames are captured at a rate dependent on the device’s hardware capacity, and the data are used to generate real-time pose estimations. The pose estimation data and the raw inertial sensor data form a comprehensive dataset for activity recognition. This multimodal dataset enhances the robustness of the model by incorporating both positional and movement data. If not required, video data can be excluded, retaining only pose estimation and/or inertial sensor data for analysis.

All recorded data, including metadata, inertial sensor data, and pose estimation data, are synchronized and formatted into a consistent dataset. The deep learning model is adept at handling this multivariate time series data, applying neural network architectures to extract relevant features directly from the raw sensor inputs. This methodology leverages the strength of deep learning to analyze complex patterns in raw data, facilitating accurate activity classification without the need for traditional feature engineering or extensive preprocessing steps.

#### 3.3.1. Pose Estimation

Pose estimation is a computer vision technique for detecting human figures and their corresponding poses from image and video data (OpenPose [59]). In HARE, we harness the video feed from the same smartphone that collects IMU data to perform pose estimations. Pose estimation is performed in real-time, ensuring immediate feedback based on the video input. This simultaneous use of a single device for both video and IMU data collection ensures synchronous data capture and aids in maintaining data integrity. We employ MoveNet Thunder’s pre-trained TFLite model for pose estimation, which processes each frame to generate key body joint landmarks [54]. The model’s output comprises 17 keypoints in 2D space, representing critical body locations such as the ankles, knees, hips, wrists, elbows, shoulders, and certain facial features.

To facilitate real-time performance, video frames undergo preprocessing to meet the model’s input specifications. This includes normalizing pixel values, resizing frames to the required resolution, and maintaining a consistent frame rate, which is essential for the subsequent time series analysis. The deep learning algorithm employed by the MoveNet model identifies and tracks keypoints, applying a confidence threshold to ensure landmark detection accuracy. Any occlusions or instances of partial visibility are managed through post-processing techniques designed to smooth the temporal trajectories of the keypoints.

**Figure 3:**
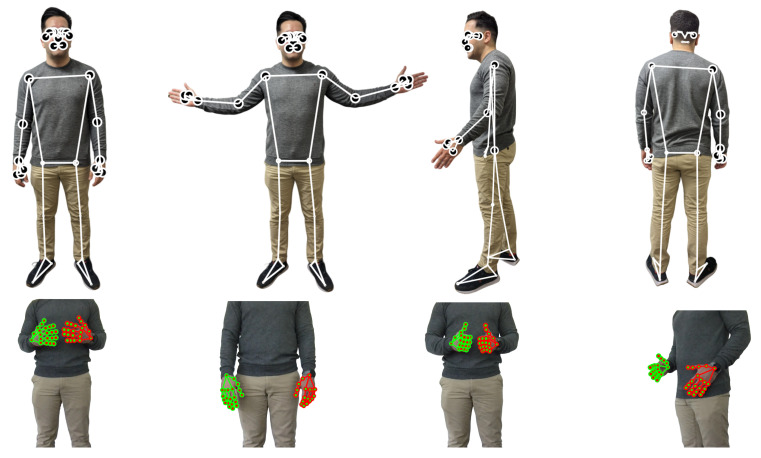
HARE’s adaptability to different use cases is demonstrated by leveraging diverse landmark estimation models. The top row shows examples of 2D pose estimations, while the second row illustrates hand landmarks with specific annotations (e.g., right hand with red circles and green connection lines, left hand with green landmarks and red connection lines). By utilizing different models, HARE can accommodate a wide range of applications.

HARE is designed to enhance pose estimation capabilities by enabling the integration of additional pre-trained models suitable for various use cases. For instance, MediaPipe offers a suite of pre-trained models that include face detection, face mesh, iris landmarking, and hair segmentation, which can be leveraged to extend the functionality of our application [60]. As demonstrated in Figure 3, our current framework incorporates hand and pose landmark estimations, showcasing HARE’s adaptability to diverse application requirements.

#### 3.3.2. Multimodal Data Integration and Synchronization

Integrating pose estimation sequences and IMU data is pivotal in HARE. These data sequences differ in their informational content. Pose sequences, derived from video data captured by the same smartphone’s camera used for IMU data collection, store relative positions, detailing spatial relationships of specific body points. In contrast, IMU data comprise position-independent sensor readings, providing a complementary perspective. Fusing these data types results in an enriched informational set.

Both data types are treated as time series, allowing their integration to be treated as a multivariate time series. This is suitable as input for a deep learning model designed to classify activities from sensor data alone. To achieve this integration, the pose estimation data undergo specific preparation. Pose sequences are recorded at a frequency of 10 Hz and are synchronized with the higher-frequency IMU data, typically collected at 60 Hz. An interpolated data series of pose estimation for each sensor datum’s timestamp is created for synchronization. More significant gaps in the data are addressed using value imputation [61], a method where missing values are replaced with a predetermined value (−1 in our case). This strategy ensures that the temporal alignment between the pose and IMU data is maintained, facilitating the creation of a cohesive and synchronized dataset.

Additionally, keypoints in all pose data series are centralized and reduced to 12 keypoints, focusing on the most informative points to eliminate redundancy. This reduction, combining stationary keypoints such as those from the head and hips, streamlines the dataset for efficient processing and analysis.

The final step involves concatenating the resultant pose sequences along the time axis with the IMU sequences to create a comprehensive multimodal input. This integration is crucial for the HARE system’s effectiveness, ensuring that time-aligned multimodal data enhances the activity recognition’s accuracy and reliability. Through this process, HARE leverages the full spectrum of data modalities, providing a robust foundation for the application’s deep learning models.

#### 3.3.3. Optimal Sensor Placement

The outcome of the pose estimations yields 2D keypoints for each timestamp. Since the position data exhibit a causal relationship with acceleration, we consider each keypoint an accelerometer. Consequently, we interpret each keypoint as a potential location for sensor placement [26].

To calculate the optimal sensor placement, we employ a three-phase algorithmic procedure. In the first phase, we pre-process the selected pose estimations by consolidating specific keypoints. Head-related and hip keypoints are replaced with a single average keypoint, while the remaining 12 keypoints are centralized around a center of mass point (0.5, 0.5) within each data series. To align with real-life scenarios, we reduce the number of keypoints to five, selecting the wrists, ankles, and pelvis. This subset of keypoints has been shown to provide rich information for full-body pose estimation [62]. The head is excluded from sensor placement as it is considered less relevant to the studied movements.

In the second step, we introduce a cross-validated feature metric, denoted as $D_{k}$. It is the core of our methodology and derived from the concept of cosine similarity, a measure traditionally used to determine the angle between two non-zero vectors in a multi-dimensional space. Each vector represents the potential sensor data over time for a given keypoint. A lower cosine similarity, or conversely, a higher cosine distance, signifies a more significant movement distinction between activities, which is desirable for sensor differentiation and placement optimization. The vectors, denoted as $A_{k}^{i}$, capture the positional data over time for each activity and keypoint subset and are assessed for their dissimilarity to determine the ideal sensor locations. To clarify, in the expression $A_{k}^{i}$:

- *k* refers to the *k*-th keypoint, which is a potential position for sensor placement.
- *i* represents the *i*-th activity out of the total number of different activities present in the dataset.
- Each vector $A_{k}^{i}$ embodies the data for a specific activity as observed at the *k*-th keypoint.

Each activity requires a minimum sequence length of 500 data points (corresponding to a 50-s recording at 10 Hz), making it possible to run efficiently on a mobile device. The $D_{k}$ metric is computed as follows:

(1) $$\begin{array}{c} D_{k}:=\sum_{i=1}^{n-1}\sum_{j=i+1}^{n}\left|1-\frac{A_{k}^{i}\cdot A_{k}^{j}}{∥A_{k}^{i}∥∥A_{k}^{j}∥}\right|. \end{array}$$

In this formula:

- The term $A_{k}^{i}\cdot A_{k}^{j}$ represents the dot product of the activity vectors for two different activities *i* and *j* at the same keypoint *k*.
- The denominator $∥A_{k}^{i}∥∥A_{k}^{j}∥$ is the product of the norms of these vectors, ensuring that the cosine similarity is normalized.
- A higher $D_{k}$ value suggests greater dissimilarity between activities at a given keypoint.

In the concluding phase of our sensor placement optimization, we prioritize sensor locations based on the $D_{k}$ metric, sorting them in descending order of their values. Sensor locations with higher $D_{k}$ values are considered more optimal due to their greater movement distinctiveness. These prioritized placements are then clearly presented within a dialog box interface for user selection or further analysis.

For scenarios that require the deployment of multiple sensors, we facilitate the evaluation of sensor pairings through vector concatenation. This process involves the sequential joining of activity-specific vectors from each potential sensor location. For instance, to assess the combination of a right wrist and pelvis sensor, we concatenate the activity vectors from the right wrist with those from the pelvis, creating a new, extended vector representing the activity data for this dual sensor setup. Such composite vectors enable direct comparison across various two-sensor combinations, helping to determine the most effective pairings for capturing distinct activity patterns.

By utilizing a vision-based virtual sensor approach, we can determine the optimal sensor placement more efficiently than physical IMUs and subsequent model training and evaluation. Thus, having physical sensors or collecting IMU data is not required. Instead, existing or self-recorded videos of targeted activities suffice to receive sensor placement recommendations.

### 3.4. Dashboard

Ensuring data quantity and quality is crucial for the training process of an ML model. Therefore, HARE provides a dashboard for data exploration and analysis. As shown in Figure 2c, it provides statistics and filtering per person and activity, which can also be used for process mining [63], e.g., nurses could analyze their data on how much time they have spent on specific activities compared to other nurses. Furthermore, it can help to optimize the execution time or rearrange the order of activities.

### 3.5. Real-Time Activity Recognition

We integrated a real-time activity recognition TFLite model, a lightweight, fast, cross-platform, open-source ML framework specifically designed for mobile and IoT [54]. There is no requirement for the model’s input size. Therefore, it is possible to train and infer models based on either a single modality or through the combination of IMU, video, and pose estimation. TFLite also facilitates on-device ML. We offer two options to train a model, (1) offline and (2) on-device.

#### 3.5.1. Offline Training

Offline training refers to the option to train a model outside the app [64]. Using the WebDAV [65] protocol, recorded data can be uploaded to an external server by providing the settings.

#### 3.5.2. On-Device Training

The second option includes the possibility of training a model on-device [66]. This enables the model to be improved on individual data, thus increasing the accuracy in new settings. On-device training also enables updating a model without transferring data to external devices, ensuring user privacy. Additionally, we do not require users to use the latest offline model. The efficacy of on-device training depends on the use case and can exhibit considerable variation, with training durations ranging from mere seconds to several minutes. We advise pre-training the model offline and fine-tuning it on the device to avoid prolonged on-device training times. Similar to model inference, on-device training also causes rapid battery depletion. Although training a model from scratch on-device utilizing the aforementioned features is feasible, this methodology requires a considerable amount of training data and time, even when executed on high-powered servers. Hence, retraining a pre-existing model specifically for on-device applications is advised.

We provide pre-trained classification models for different use cases and allow users to fine-tune the model using transfer learning after collecting and labeling small amounts of new data. In that case, the deep learning model’s layers will be frozen, excluding the last, and trained for a defined number of epochs. After completing a training run on a device, the model updates the set of weights in storage that can be saved in a checkpoint file for later use. As an interactive outcome, we display a performance list for each activity in a confusion matrix before and after on-device training. In doing so, we facilitate a human-in-the-loop [67] process, which informs the study conductor of poorly recognized activities. They can then decide to collect more data for these activities to improve model accuracy.

## 4. Experimental Evaluation: Nursing Activity Recognition

To evaluate the functionality and advantages offered by HARE, we collected data on nursing activities under the guidance of a professional nurse. To showcase its versatility and effectiveness, we conducted an experiment specifically focusing on nursing activities. Nursing activities were chosen due to their diverse and complex nature, demanding precise and efficient data collection and classification. By deploying HARE in this real-world scenario, we effectively demonstrate its capability to address the challenges associated with data collection, classification, and privacy preservation. Moreover, this experiment is an illustrative example of how our framework can be adapted to different domains and applications, highlighting its potential to contribute to the HAR field.

### 4.1. Data Description

**Figure 4:**
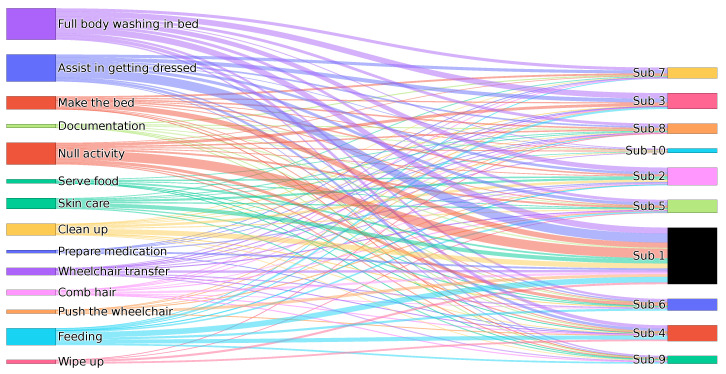
Sankey diagram showing the distribution of activities performed by Subjects 1 to 10 (denoted as Sub 1–Sub 10).

For the purpose of this study, we engaged in data collection employing a set of five Xsens DOT sensors positioned at key anatomical locations: specifically, the left wrist, right wrist, pelvis, left ankle, and right ankle. These sensors were configured to deliver data at an output rate of 60 Hz, yielding an expansive dataset. Within each sensor, we obtained a rich stream of information, consisting of 14 sensor streams per sensor (70 streams overall). These streams encompassed a spectrum of data, including four-dimensional quaternion values, four-dimensional angular velocity data derived from the quaternion values, three-dimensional acceleration values, and three-dimensional magnetic field values. In our experiments, the video recording for pose estimation was conducted in a lab environment measuring approximately 4 m × 7 m. The mobile device used for recording was positioned at a distance of approximately 2 to 3 m from the subject. This distance was found to be optimal for capturing the subject’s full range of motion while maintaining the quality of the recorded video. It is important to note that the pose estimation model employed in our app normalizes the subject to the screen center. Therefore, variations in the distance between the mobile device and the subject do not significantly impact the pose estimation results. This normalization ensures consistent pose estimation accuracy regardless of minor changes in the recording setup. Our dataset comprises 13 different activities by ten subjects. The result was a compilation of 51 recordings, resulting in nearly 8 h of documented data. The distribution of subjects across various activities is illustrated in Figure 4. Further, the data collection, classification, and privacy preservation techniques presented in this work are adaptable. Regardless of the specific sensor types, they can be applied across various HAR scenarios.

### 4.2. Results

Offline training involved the utilization of three deep learning models. To enhance the performance, we employed hyperparameter optimization through grid search [68], resulting in a learning rate of 1 × 10^−4^ and an input size set at 600×70. This input size corresponds to a 10-s window captured at a sampling rate of 60 Hz. Each model integrated a preprocessing step designed to handle missing data, complemented by incorporating a batch-normalization layer, which standardized inputs within each feature row. The output layer of these models featured a dense softmax layer configured to align with the number of activity classes. During the training process, we harnessed the Adam optimization method [69]. For our multi-class classification problem, we adopted the following categorical cross-entropy loss function:

$$\begin{array}{c} L=-\log\left(\frac{e^{s_{p}}}{\sum_{j}^{C}e^{s_{j}}}\right) \end{array}$$

Here, *C* represents the set of classes, *s* denotes the vector of predictions, and $s_{p}$ indicates the prediction for the target class.

The architecture of the three models is defined below.

1. The CNN-LSTM model is composed of six layers. The input layer is followed by two convolutional layers, two LSTM [70] layers, and the output layer.
2. The ResNet model is composed of 11 layers. Next to the input and output layers, it has a repeated sequence of three convolution layers followed by a batch normalization layer and an activation layer [71].
3. The DeepConvLSTM model is composed of eight layers. After the input layer, it is followed by four consecutive convolution layers and two LSTM layers before the softmax classifier [72].

For evaluation, we also used three different cross-validation techniques, namely, (1) k-fold cross-validation on time windows of length 600 with k=5; (2) leave-recordings-out cross-validation: One recording corresponds to starting and ending a recording in one go. In our study, the recordings are between ∼46 s and ∼1249 s, and we used an 80:20 train-test ratio. One recording can contain only one specific activity or multiple activities performed multiple times. This validation technique reflects the model’s performance when used within HARE; (3) lastly, we evaluate leave-one-subject-out cross-validation.

As shown in Table 2, the ResNet model performance is best for k-fold cross-validation, whereas the CNN-LSTM model performs best on the leave-one-{recording/subject}-out cross-validation metric.

**Figure 5:**
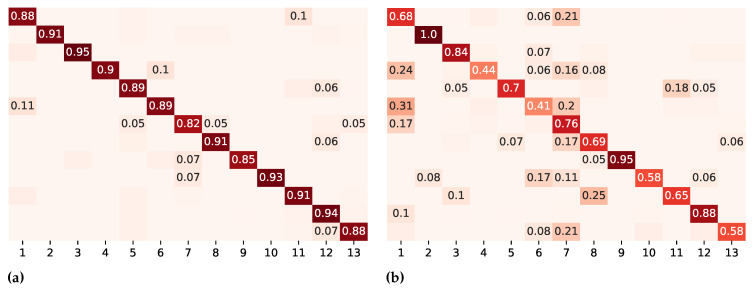
Confusion matrices on different evaluation metrics for DeepConvLSTM. Numbers below 0.05 are left out. The color intensity represents the degree of similarity between predicted and actual values: darker colors along the diagonal represent accurate predictions (values closer to 1), while lighter colors off the diagonal indicate misclassifications (values closer to 0). The color intensity serves as a visual cue, emphasizing the model’s performance in correctly predicting different classes. (**a**) k-fold cross-validation. (**b**) Leave-recordings-out cross-validation. Legend: 1 = assist in getting dressed; 2 = full body washing in bed; 3 = feeding; 4 = make the bed; 5 = clean up; 6 = skin care; 7 = push the wheelchair; 8 = wheelchair transfer; 9 = comb hair; 10 = wipe up; 11 = prepare medication; 12 = serve food; 13 = documentation.

From the data in Figure 5a, we see that the model on k-fold cross-validation is able to distinguish well between activities. The results of the confusion matrix for leave-recordings-out cross-validation are set out in Figure 5b. It is apparent from this matrix that some activities are mixed up when trained on recordings. *Making the bed*, e.g., has a low accuracy and is confused with *assist in getting dressed*. Similarly, *skin care* is confused with *assist in getting dressed* or *push the wheelchair*.

#### 4.2.1. Multimodal Activity Recognition

We employed the CNN-LSTM model architecture described in Section 4.2 for the multimodal classification approach. This decision was informed by the model’s performance in our baseline studies, particularly in leave-recordings-out and leave-one-subject-out evaluations. These metrics are crucial for assessing the model’s adaptability to new data and subjects, mirroring the anticipated real-world application of our system. Additionally, the CNN-LSTM architecture represents a tailored adaptation in our research, differing from standard models cited in the literature. Focusing on this architecture allowed us to optimize our computational resources and delve deeper into the potential of multimodal data integration. This architectural approach was used to combine the inertial data and pose estimations. The results for each modality combination in the nursing dataset are presented in Table 3. It is apparent from this table that combining both IMU and pose estimation data consistently yielded the best performance, while relying solely on pose estimation data resulted in the poorest performance across all modalities.

The findings presented in Table 4 validate our observations, as they are consistent with results obtained from a second dataset focusing on simple activities in daily living, including walking, walking upstairs, walking downstairs, standing, sitting, and lying. It is worth noting that, in each investigated case, the pose estimation modality’s performance is noticeably lower than other modalities.

#### 4.2.2. On-Device Learning

**Figure 6:**
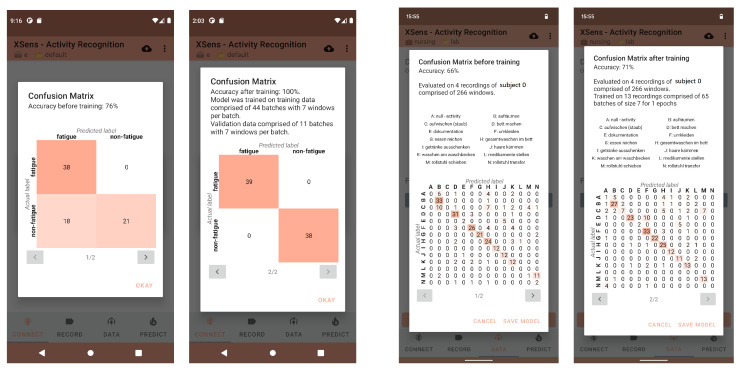
Comparison of the classification results before (first and third picture) and after (second and last picture) on-device transfer learning for two different datasets depicted in a confusion matrix within HARE. The first two images show the gait analysis dataset, whereas the last two illustrate the results for the nursing dataset.

To evaluate the performance of our on-device training, we employed the leave-one-subject-out cross-validation method, in which we trained the model on data from all subjects except one, using this withheld subject’s data for validation. The model trained using the leave-one-subject-out cross-validation served as a reference model, which we compared with the model trained on the left-out subject’s data to evaluate the effectiveness of our on-device training approach. We employed a two-step evaluation process to assess the effectiveness of our on-device training approach. First, we used the leave-one-subject-out cross-validation method, where we trained the model on data from all subjects except one and reserved this left-out subject’s data for evaluation. The prediction accuracy obtained for the left-out subject served as a reference value. Next, we further evaluated the model’s performance on the left-out subject’s data using the leave-recordings-out cross-validation method. We split the left-out subject’s recordings into an 80:20 ratio, with 80% for training and 20% for testing. This allowed us to provide an unbiased estimate of the model’s performance and compare it with the reference value obtained from the leave-one-subject-out cross-validation. Since only four subjects performed all activities and had at least five recordings for validation, we only included them for comparison. We used 10 epochs to retrain the given model on all four subjects. On-device training, utilizing TFLite for classification, varies in duration. Given our setup with transfer learning and a batch size of 7, the training time was between 7 and 9 min for a small dataset. Table 5 shows increased classification performance for all considered models using this approach. Two additional datasets confirmed the results. For all investigated models on the OPPORTUNITY dataset [73], we achieved an average accuracy improvement over all subjects of 47% for the ResNet model, as shown in Table 6. Another dataset on gait analysis [74] led to improved average performance over all subjects of 49%, as highlighted in Table 7. The improved accuracy through on-device training can be further explored per activity by displaying the confusion matrix depicted in Figure 6.

#### 4.2.3. Optimal Sensor Placement

**Figure 7:**
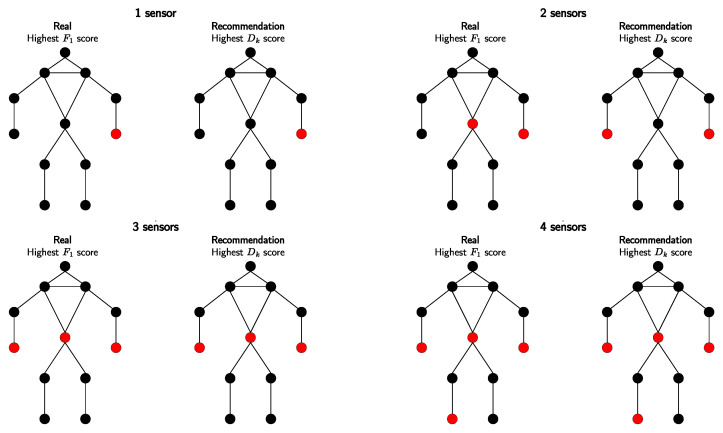
Comparing sensor placements for nursing activity monitoring: F1 (**left**) vs. $D_{k}$ analysis (**right**). Optimal positions are highlighted in red. Each image reveals the best placement corresponding to the number of sensors available.

Given five sensors, we trained a total of 31 models for all possible sensor combinations with the CNN-LSTM model. The F1 score was different depending on the sensor’s location. Similarly, we obtained different results for $D_{k}$, calculated from 3000 data rows. This corresponds to 5 min at a frequency rate of 10 Hz per activity and sensor. Since we collected data from five sensors, we only considered results for all five body locations. The results for both approaches are shown in Figure 7. This figure shows that three out of four recommended sensor placements match the classification results. There is only a slight difference in the placement of two sensors. Determining the optimal sensor placement with our approach took only 185 s compared to the 27 h of model training with the classical approach.

We also used the deep inertial poser (DIP) dataset, which provides valuable insights for evaluating our optimal sensor placement approach [62]. The DIP dataset is a collection of real IMU data augmented with corresponding 3D pose estimations based on the skinned multi-person linear (SMPL) model [75].

The SMPL model is a foundation for representing human body pose and shape variations. It is defined by a set of pose parameters θ and shape parameters β, which capture joint rotations and body shape, respectively. The model defines a mapping from the pose and shape parameters to the 3D coordinates of the body vertices, denoted as *V*. This mapping is represented by the function V=M(θ,β) and includes a total of 6890 vertices.

**Figure 8:**
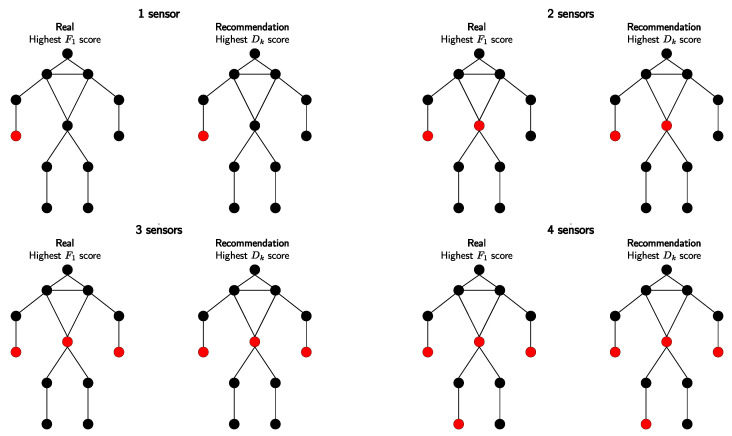
Comparing sensor placements for DIP-IMU: F1 (**left**) vs. $D_{k}$ analysis (**right**). Optimal positions are highlighted in red. Each image reveals the best placement corresponding to the number of sensors available.

Our proposed approach leverages both IMU readings and the corresponding 3D pose estimations of arbitrary body points. Due to the limited availability of datasets that include synchronized IMU data and 3D pose estimations, the applicability of our approach is currently constrained to a few datasets, like the DIP dataset. The dataset consists of approximately 90 min of real IMU data collected from 10 subjects wearing 17 Xsens sensors. The dataset encompasses 64 sequences with 330,000 time instants. Participants were instructed to perform various motions falling into five different categories. These categories include controlled motion of the extremities (e.g., arm and leg movements) in (1) locomotion, (2) upper body, (3) lower body, (4) freestyle, and (5) interaction. The dataset comprises 13 activities, providing a diverse range of motions for validation and analysis. The dataset’s modalities and division into categories make it particularly interesting and suitable for our approach. We chose the same five positions (wrists, ankles, and pelvis) to track the movements in 3D. As illustrated in Figure 8, we achieved a total match with the trained model on this dataset.

Further, we conducted a small-scale experiment to demonstrate HARE’s sensor placement capabilities. The experiment involved five participants engaging in six different activities of daily living (ADL): sitting, walking, ascending stairs, descending stairs, standing, and lying down. Each activity was performed for ten minutes. Three sensors were placed at the right wrist, pelvis, and right ankle. This setup aimed to capture a comprehensive range of motion data for each activity. The results from our sensor placement analysis indicated that the right wrist is the optimal location for sensor placement in both approaches.

## 5. Discussion

This paper addresses the development of an application combining various HAR functionalities in a single application. Special attention is given to real-time classification, a multimodal approach, on-device training, and optimal sensor placement.

The results from the single modality analysis show that k-fold cross-validation provides the best results. This is related to the training process of the network being able to see a portion of the data from all subjects. Theoretically, leave-recordings-out can comprise the same data as leave-one-subject-out or k-fold. This depends on how the data were recorded. Therefore, it is not possible to conclude about the model’s performance, depending on the recording’s composition. The given result for leave-recordings-out may be explained by the fact that the model has not seen recorded data on all subjects or activities per subject, which can be assumed from the lower accuracy compared to k-fold cross-validation. This is also supported by the performance on each activity, presented in Figure 5b. Certain activity misclassifications, such as classifying *making the bed* as *assist in getting dressed* and vice versa, are likely to be related to similar movement patterns. Differences between activities *skin care* and *assist in getting dressed* are also related to similar movements. On the other hand, the activity *full body washing in bed* is always predicted correctly. This result may be explained by the fact that this activity was the most recorded.

Overall, the ResNet model performed best on k-fold cross-validation. The shallower model, CNN-LSTM, has more trainable parameters and performs best on the other two evaluation metrics. The discrepant results for leave-one-subject-out cross-validation in Table 2 and offline training in Table 5 are due to the subjects considered. We only included the four subjects that performed all activities and had at least five recordings to make the evaluation possible. However, we could show that this can be compensated by on-device training.

While multimodal classification approaches exist, the unique advantage of HARE is its integrated data collection. Unlike existing approaches that require multiple devices and subsequent synchronization, HARE simultaneously collects pose estimation and IMU data. This approach simplifies the process while ensuring seamless data integration for improved classification accuracy. However, the observed performance boost is relatively modest. Two potential factors may contribute to this outcome. Firstly, the variations in camera angles during data acquisition could impact the results. When recordings are captured from a side view, the keypoints tend to be closer together in the 2D space, posing a challenge for accurate classification. The viewing angle of the camera, therefore, plays a crucial role in the overall performance. Secondly, the absence of pose estimations under certain conditions could affect the results. When the camera fails to capture the entire body or significant portions of it, obtaining accurate pose estimations becomes infeasible. Out of the 51 recordings examined, ten recordings lack pose estimation data due to these limitations. These factors highlight the complexities and limitations inherent in combining IMUs and pose estimation data for improved classification. Understanding the influence of camera angles and addressing challenges related to pose estimation under various conditions are essential considerations for further enhancing the performance of our multimodal approach.

The cross-validated feature metric $D_{k}$ represents an innovative aspect of our research, differing significantly from traditional sensor placement strategies. By leveraging the collected pose estimation data, we optimize sensor placement more efficiently, eliminating the need for additional devices or time-consuming model training processes. It effectively captures the significance of hand movements and shows the successful performance of multiple sensor combinations. Interestingly, the approach yields accurate results even when a sensor detects minimal motion, such as the pelvis sensor. These findings align with those obtained from trained models, suggesting that the sensors act as counterparts, with one serving as a root or reference point. However, it is important to note that using different ML models can lead to varying outcomes. Therefore, it becomes challenging to attribute the minimal difference between the two sensors to the specific model used. Different ML models may produce slightly different results, and further investigation is necessary to ascertain the relationship between sensor differences and model performance. Overall, the consistency between the cross-validated feature metric and trained models highlights the effectiveness of our approach in determining optimal sensor localization. The collaborative nature of the sensors and the influence of different ML models contribute to the subtle results observed, underlining the complexity of sensor placement optimization in human activity recognition.

### Limitations

Our approach comes with some limitations. Currently, we only offer the connection with the Xsens DOT sensors, and HARE is only available for the Android operating system. When forming pose estimations, distortions in the image can occur quickly if there are objects in front of the person or if the focus is shifted. This leads to low confidence values and, thus, gaps in data collection. Consequently, this would corrupt both a multimodal approach and the optimal determination of sensor positions. Furthermore, our method employs a 2D pose estimation technique that does not consider depth mapping. Consequently, when the person being observed turns or the recording angle changes, there may be inaccuracies in estimating the person’s distance. While our optimization method for sensor placement has demonstrated its effectiveness, ease of implementation, and speed, it is essential to acknowledge that our study did not include a direct comparison with other methods for optimization. The main reason for this omission is the limited availability of readily implementable alternatives for direct evaluation.

## 6. Conclusions and Outlook

We designed an on-body sensor-based HAR application system in this research study and evaluated its features. In conclusion, our work introduces several innovations and contributions to HAR. First, we streamline the data collection process and integrate a novel approach for multimodal classification using a single device. This approach represents a significant advancement over traditional methods that rely on multiple devices and complex synchronization. Second, the pose estimation technique can effectively anonymize test subjects. Third, we demonstrate the possibility of determining the optimal sensor placement without the necessity of actual IMUs. To determine the optimal placement, videos from targeting activities are sufficient. Lastly, the possibility to infer a personalized, privacy-preserving on-device trained model and a multimodal real-time classification approach on different use cases.

Our vision-based approach has demonstrated how integrating several HAR tasks into a single workflow can significantly enhance the efficiency and accuracy of HAR. We aim to further these innovations by exploring advanced pose estimation techniques, such as 3D pose estimation models, video recordings, and diverse sensor types. Future work will also address implementing and comparing other sensor optimization methods. 

## Figures and Tables

**Table 1:** Comparison of sensor-based HAR frameworks and methods.

		Helmy and Helmy [45]	Añazco et al. [46]	Köping et al. [47]	Mairittha et al. [48]	Gudur et al. [38]	Xia and Sugiura [25]	Kim et al. [49]	Ijaz et al. [50]	Google AR API [51]	Apple Motion [52]	Microsoft Kinect [53]	HARE (Ours)
Data Collection	External Sensor Connectivity	✗	✓	✓	✗	✗	✗	✗	✗	✗	✗	✗	✓
	Multimodal Recording	✗	✗	✓	∼	✗	✗	✗	✓	✗	✗	✗	✓
	Extendability *	∼	✗	✓	✓	✓	✗	✗	✗	✗	✗	✗	✓
Classification	Real-time	✓	✓	✓	∼	✗	✗	✗	✗	✗	✗	✓	✓
	Multimodality	✗	✗	✓	✗	✗	✗	✗	✓	✗	✗	✗	✓
Labeling	Refinement	✗	✗	✗	✓	✗	✗	✗	✗	✓	✓	✗	✓
	Anonymization	✗	✗	✗	✗	✗	✗	✓	✗	✓	✓	✓	✓
Sensor Placement	Pose Estimation	✗	✗	✗	✗	✗	✗	✗	✗	✓	✓	✗	✓
	Optimal Position	✗	✗	✗	✗	✗	✓	✗	✗	✗	✗	✗	✓
ML-based Feedback	On-Device Training	✗	✗	✗	✗	✓	✗	✗	✗	✗	✗	✗	✓
	Human-in-the-Loop	✗	✗	✗	✓	✓	✗	✗	✗	✗	✗	✗	✓
	Extended Logging ${}^{¶}$	✗	✗	✗	✗	✗	✗	✗	✗	✗	✓	✗	✓

∼: Partially supported. *: Adaptable to different domains through extending the landmark model. ${}^{¶}$: Additional statistics per person and activity.

**Table 2:** We compare the baseline results of three deep learning models (CNN-LSTM, ResNet, and DeepConvLSTM) with Accuracy (higher is better) and F1 score (higher is better) using three cross-validation methods (k-fold, leave-recordings-out, and leave-one-subject-out) with five sensors. The standard deviation is reported with the ± symbol. We embolden the model per column.

Model	k-Fold		Leave-Recordings-Out		Leave-One-Subject-Out	
	**Accuracy**	**F1**	**Accuracy**	**F1**	**Accuracy**	**F1**
CNN-LSTM	0.832 (±0.01)	0.830 (±0.02)	**0.632 (±0.03)**	**0.630 (±0.02)**	**0.565 (±0.05)**	**0.556 (±0.04)**
ResNet	**0.864 (±0.01)**	**0.864 (±0.01)**	0.600 (±0.02)	0.603 (±0.03)	0.531 (±0.05)	0.527 (±0.03)
DeepConvLSTM	0.735 (±0.02)	0.730 (±0.04)	0.563 (±0.05)	0.565 (±0.05)	0.525 (±0.06)	0.515 (±0.04)

**Table 3:** Classification results obtained for three different input data modalities: IMU, pose estimation, and the combination of IMU and pose estimation (IMU+pose estimation), using three evaluation methods: k-fold, leave-recordings-out, and leave-one-subject-out. The experiments were conducted to classify high-level activities constituting complex activities in nursing. We report the same metrics for each combination of input data and evaluation methods. We embolden the model per column.

Input Data	k-Fold		Leave-Recordings-Out		Leave-One-Subject-Out	
	**Accuracy**	**F1**	**Accuracy**	**F1**	**Accuracy**	**F1**
IMU	0.823 (±0.03)	0.822 (±0.04)	0.615 (±0.04)	0.611 (±0.06)	0.489 (±0.05)	0.474 (±0.06)
Pose Estimation	0.421 (±0.07)	0.361 (±0.03)	0.424 (±0.06)	0.410 (±0.07)	0.359 (±0.05)	0.281 (±0.08)
IMU + Pose Estimation	**0.838 (±0.01)**	**0.836 (±0.02)**	**0.641 (±0.01)**	**0.638 (±0.03)**	**0.501 (±0.02)**	**0.478 (±0.03)**

**Table 4:** Classification results obtained for three different input data modalities: IMU, Pose Estimation, and the combination of IMU and Pose Estimation (IMU+pose estimation), using three evaluation methods: k-fold, leave-recordings-out, and leave-one-subject-out. The experiments were conducted to classify low-level activities constituting simple activities of daily living. We embolden the best model per column.

Input Data	k-Fold		Leave-Recordings-Out		Leave-One-Subject-Out	
	**Accuracy**	**F1**	**Accuracy**	**F1**	**Accuracy**	**F1**
IMU	0.912 (±0.03)	0.912 (±0.02)	0.895 (±0.02)	0.893 (±0.02)	0.860 (±0.04)	0.854 (±0.03)
Pose Estimation	0.533 (±0.07)	0.465 (±0.09)	0.550 (±0.06)	0.532 (±0.07)	0.569 (±0.08)	0.511 (±0.06)
IMU + Pose Estimation	**0.920 (±0.01)**	**0.917 (±0.01)**	**0.902 (±0.02)**	**0.898 (±0.01)**	**0.887 (±0.01)**	**0.871 (±0.02)**

**Table 5:** The classification results for different models with offline and on-device training are compared. We show three different models (CNN-LSTM, ResNet, and DeepConvLSTM) with both offline and on-device training for the nursing activity dataset. The best scores are emboldened row-wise per metric. We report the same metrics for each combination of input data and evaluation methods. We embolden the best model per column.

Model	Offline Training		On-Device Training	
	**Accuracy**	**F1**	**Accuracy**	**F1**
CNN-LSTM	0.352 (±0.07)	0.310 (±0.06)	**0.527 (±0.02)**	**0.492 (±0.03)**
ResNet	0.487 (±0.04)	0.473 (±0.06)	**0.489 (±0.03)**	**0.495 (±0.04)**
DeepConvLSTM	0.320 (±0.08)	0.193 (±0.09)	**0.416 (±0.04)**	**0.237 (±0.05)**

**Table 6:** The classification results for different models with offline and on-device training are compared. We show three different models (CNN-LSTM, ResNet, and DeepConvLSTM) with both offline and on-device training for the OPPORTUNITY dataset. The best scores are emboldened row-wise per metric.

Model	Offline Training		On-Device Training	
	**Accuracy**	**F1**	**Accuracy**	**F1**
CNN-LSTM	0.644 (±0.08)	0.612 (±0.09)	**0.745 (±0.09)**	**0.703 (±0.07)**
ResNet	0.493 (±0.12)	0.487 (±0.11)	**0.727 (±0.10)**	**0.671 (±0.12)**
DeepConvLSTM	0.598 (±0.09)	0.599 (±0.09)	**0.654 (±0.07)**	**0.615 (±0.06)**

**Table 7:** The classification results for different models with offline and on-device training are compared. We show three different models (CNN-LSTM, ResNet, and DeepConvLSTM) with both offline and on-device training for the gait analysis dataset. The best scores are emboldened row-wise per metric.

Model	Offline Training		On-Device Training	
	**Accuracy**	**F1**	**Accuracy**	**F1**
CNN-LSTM	0.607 (±0.12)	0.598 (±0.11)	**0.832 (±0.16)**	**0.821 (±0.13)**
ResNet	0.580 (±0.13)	0.576 (±0.12)	**0.863 (±0.19)**	**0.847 (±0.15)**
DeepConvLSTM	0.578 (±0.12)	0.530 (±0.14)	**0.817 (±0.13)**	**0.813 (±0.12)**

## Data Availability

The data from the feasibility study are accessible via Nextcloud [76]. The code for the application, including all used models, is shared on GitHub [77].
